# Supplementary material for: Inter-trial effects in visual pop-out search: Factorial comparison of Bayesian updating models
Source: PLoS Comput Biol. 2018 Jul 30;14(7):e1006328. doi: 10.1371/journal.pcbi.1006328 (PMC6091979; doi:10.1371/journal.pcbi.1006328)
Supplement: S1 Text — (DOCX) [file pcbi.1006328.s001.docx]

## S1 Text: Statistical tests of error rates

Post-hoc comparisons of the error rates among blocked target frequencies revealed that fewer errors were made in high- as compared to medium- and low-frequency blocks*,* $t\left( 11 \right)=1.7,p>0.3,BF=1.2$ , and, respectively, $t\left( 11 \right)=3.72, p<0.01, BF=248$ (Bonferoni-corrected p-value), and in medium as compared to low-frequency blocks, $t\left( 11 \right)=3.21, p<0.05,BF=53$ (Bonferoni-corrected p-value), in Experiment 1; and similarly in high- relative to medium- and low-frequency blocks, $t(11)=2.07,p=0.15,BF=16$, and, respectively, $t(11)=4.92,p<0.001,BF=228$ (Bonferoni-corrected p-value), and in medium- relative to low-frequency blocks, $t\left( 11 \right)=2.85, p<0.05,BF=7.9$ (Bonferoni-corrected p-value), in Experiment 2.

There was no interaction between target condition and frequency in either Experiment 1 or Experiment 2, $F(2,22)=0.83,p=0.45,BF=0.24$, and, respectively, $F(1.28,14.04)=0.76$ (Huynh-Feldt Corrected degrees of freedom), $p=0.43,BF=0.29$ – suggesting the effect of the frequency of a condition within a block is independent of the target stimuli.
