# Supplementary material for: Inter-trial effects in visual pop-out search: Factorial comparison of Bayesian updating models
Source: PLoS Comput Biol. 2018 Jul 30;14(7):e1006328. doi: 10.1371/journal.pcbi.1006328 (PMC6091979; doi:10.1371/journal.pcbi.1006328)
Supplement: S2 Text — (DOCX) [file pcbi.1006328.s002.docx]

## S2 Text: Statistical tests of mean RTs

In Experiment 1, RTs were significantly faster in the high-frequency compared to both the low- and medium-frequency blocks [$t\left( 11 \right)=3.96,p<0.01,BF=5.57*{10}^{4}$, and, respectively, $t\left( 11 \right)=3.88,p<0.01,BF=32.6$ (Bonferroni-corrected p-values)], while there was no significant difference between the medium- and low-frequency blocks, [$t(11)=0.086,p>0.9,BF=0.22$]. Similarly, in Experiment 2, RTs were significantly faster in the high-frequency compared to the low- and medium-frequency blocks [$t\left( 11 \right)=7.72,p<0.001,BF=2.78*{10}^{5}$, and, respectively, $t\left( 11 \right)=3.66,p<0.01,BF=90$ (Bonferroni-corrected p-values)], and also faster in the medium- compared to the low-frequency block [$t\left( 11 \right)=4.06,p<0.01,BF=455$ (Bonferroni-corrected p-value)].

Bayesian repeated-measures ANOVAs showed that there was no difference between trials with color- and orientation-defined targets in Experiment 2, $F(1,11)=0.45,p=0.52,BF=0.26$. Interestingly, there was no interaction between target condition and frequency in either Experiment 1 or 2 [$F(2,22)=2.44,p=0.11,BF=0.39$, and, respectively, $F(2,22)=0.87,p=0.43,BF=0.31$] – suggesting that the effect of the frequency is independent of the target stimuli.
