# Supplementary material for: Inter-trial effects in visual pop-out search: Factorial comparison of Bayesian updating models
Source: PLoS Comput Biol. 2018 Jul 30;14(7):e1006328. doi: 10.1371/journal.pcbi.1006328 (PMC6091979; doi:10.1371/journal.pcbi.1006328)
Supplement: S3 Text — (DOCX) [file pcbi.1006328.s003.docx]

## S3 Text: Modeling results without non-decision time

Figures A, B, and C depict the mean relative AICs, averaged across all participants, for all models without a non-decision time (NDT) component for Experiments 1, 2, and 3 respectively. Table A shows the differences in AIC between models without and with a non-decision time component. Compared to models with a non-decision time component (see the Modeling section in the main text), the average AICs were in general higher, that is, the models without a NDT component performed worse. Nevertheless, the dependence of the AIC on the other factors is very similar: in particular, the best combination of the other factor levels, for each experiment, is the same whether models without or with a NDT component are considered. One noticeable difference is that without a NDT component, the difference in AIC between models using the DDM and those using the LATER model is larger. Thus, while a NDT component is important for providing a good model fit, both for the DDM and for the LATER model, it appears that the LATER model can to some extent compensate for not including such a component through adjustment of the other parameters.


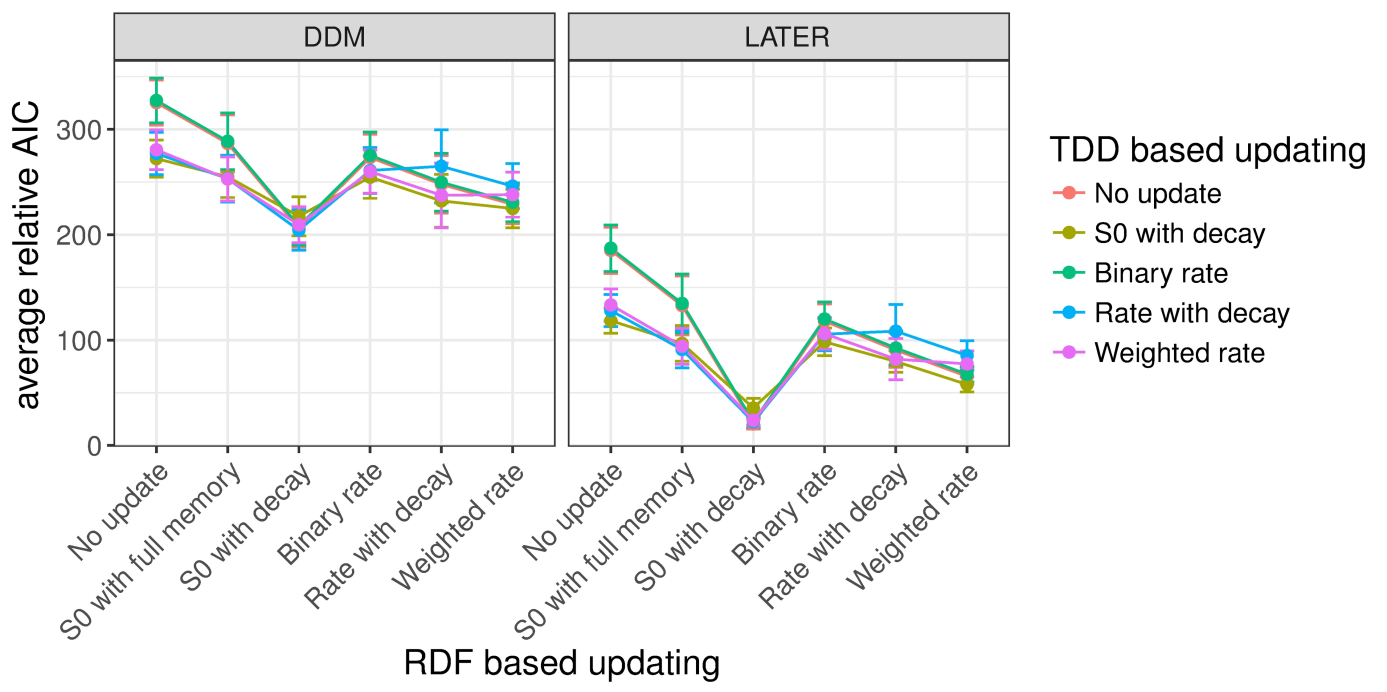


**Figure A** Mean relative AICs as a function of the tested models in Experiment 1. For each participant, the AIC of the overall best-performing model has been subtracted from the AIC for every model, before averaging across participants. Error bars indicate the standard error of the mean. The response-based updating rules are mapped on the x-axis, the dimension-based updating rules are indicated by different colors. The left panel depicts results for the DDM, the right panel for the LATER model. Only models without a non-decision time component are included in the figure.


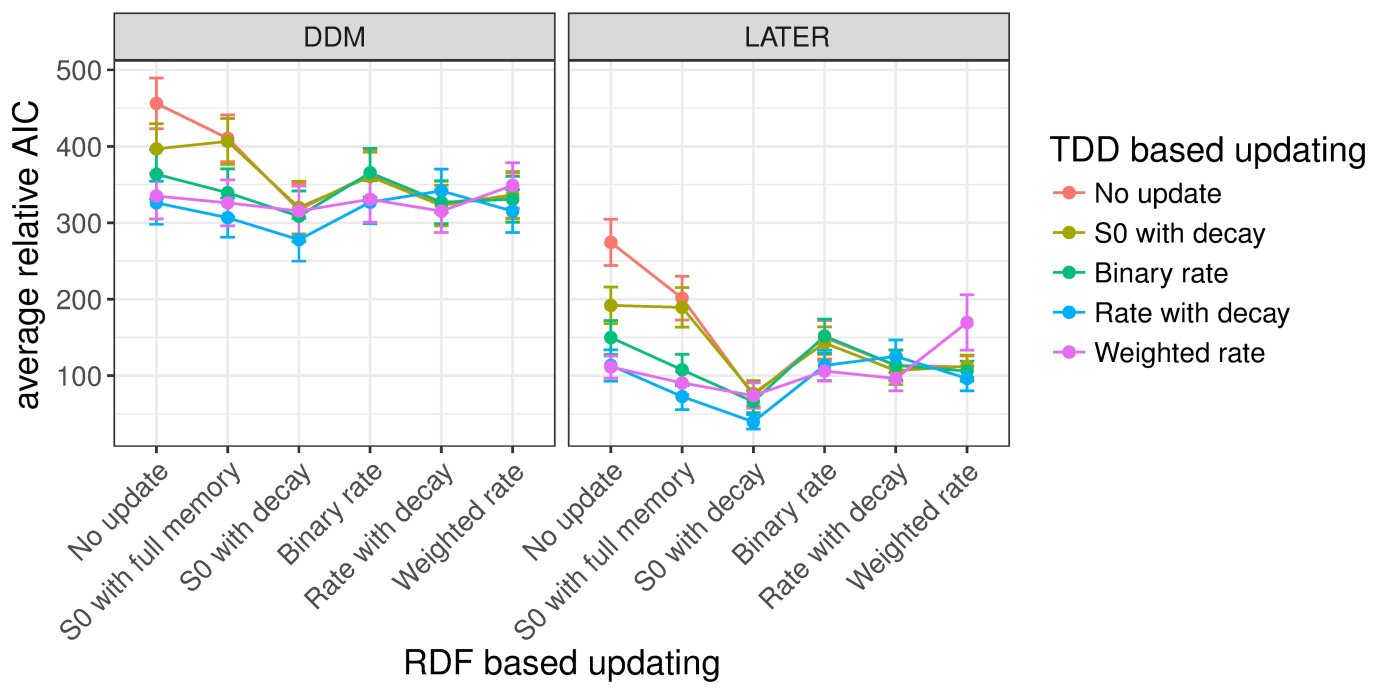


**Figure B** Mean relative AICs as a function of the tested models in Experiment 2. For each participant, the AIC of the overall best-performing model has been subtracted from the AIC for every model, before averaging across participants. Error bars indicate the standard error of the mean. The response-based updating rules are mapped on the x-axis, the dimension-based updating rules are indicated by different colors. The left panel depicts results for the DDM, the right panel for the LATER model. Only models without a non-decision time component are included in the figure.


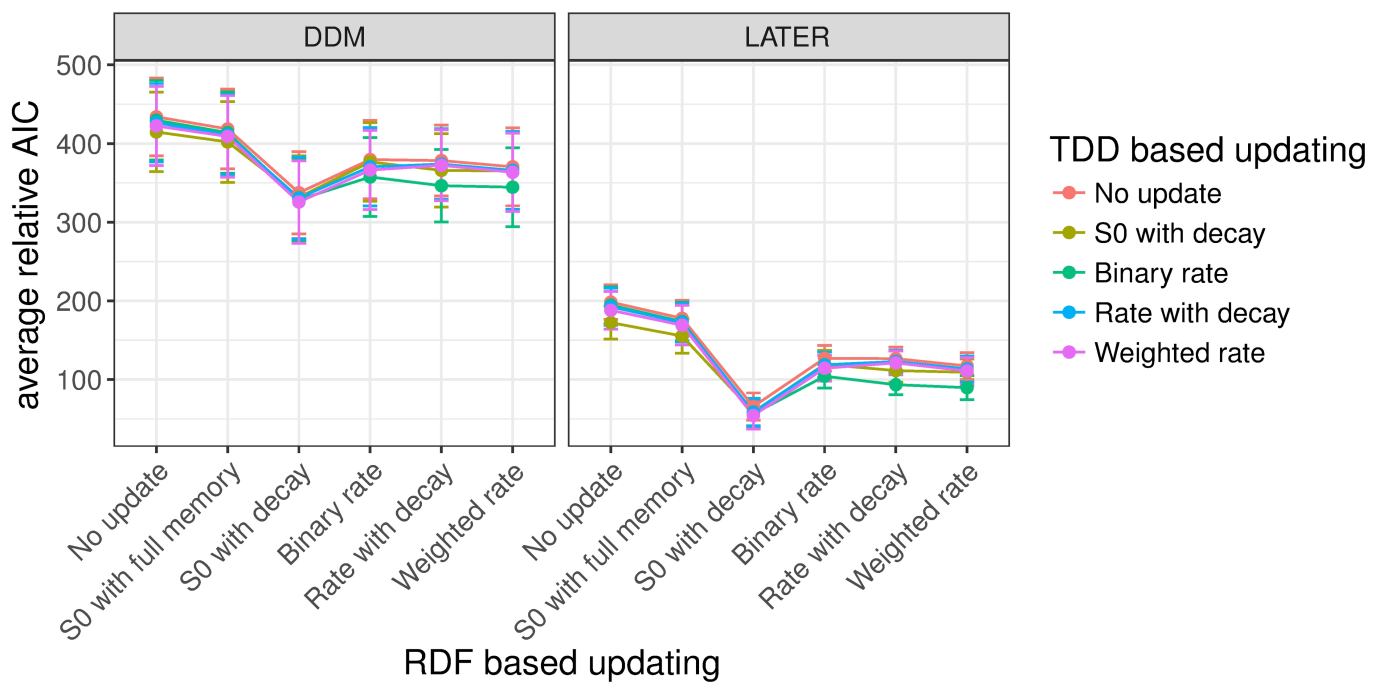


**Figure C** Mean relative AICs as a function of the tested models in Experiment 3. For each participant, the AIC of the overall best-performing model has been subtracted from the AIC for every model, before averaging across participants. Error bars indicate the standard error of the mean. The response based updating rules are mapped on the x-axis while the dimension based updating rules are mapped to different colors. The left panel contains results for the DDM while the right panel contains results for the LATER model. Only models without a non-decision time component are included in the figure.

**Table A** Means (and associated standard errors) of the differences in AICs between models without and with a non-decision time component.

|  | Experiment 1 | | Experiment 2 | | Experiment 3 | |
| --- | --- | --- | --- | --- | --- | --- |
|  | No updating | Best model | No updating | Best model | No updating | Best model |
| LATER | 6.1 (3.3) | 20.4 (4.9) | 17.6 (6.6) | 39.6 (9.5) | 34.0 (12.4) | 54.3 (17.3) |
| DDM | 115.8 (12.9) | 153.0 (13.6) | 149.1 (23.6) | 185.7 (22.4) | 199.4 (37.1) | 230.0 (38.4) |

Note: The “No updating” columns show the differences for models without any updating, while the “Best model” columns show the differences when using the updating models for which the lowest AIC was obtained.
