# Supplementary material for: Inter-trial effects in visual pop-out search: Factorial comparison of Bayesian updating models
Source: PLoS Comput Biol. 2018 Jul 30;14(7):e1006328. doi: 10.1371/journal.pcbi.1006328 (PMC6091979; doi:10.1371/journal.pcbi.1006328)
Supplement: S4 Text — (DOCX) [file pcbi.1006328.s004.docx]

## S4 Text: Model comparison based on individual participants

Another way of comparing the models is by picking the best model, in terms of AIC, for each participant and counting how often each level of each factor appears in the resulting list. Table A summarizes the results of such an analysis. The table has four sections corresponding to the different factors of our factorial model comparison: RT distribution model, non-decision time, response-based updating, and dimension-based updating. Almost all the best-fitting models were based on the LATER model, rather than the DDM – consistent with the analyses based on average AIC values above. Also, nearly all best-fitting models included a non-decision time parameter. Inclusion of a non-decision time is, of course, unsurprising for (the best-fitting) models based on the DDM. Standard versions of the LATER, by contrast, have hardly ever included a non-decision time component. Our results suggest that adding such a component improves the fit to the data sufficiently to motivate this extra parameter.

For the response-based updating, almost all the best-fitting models were based on updating of the starting point, rather than the rate. In addition, most of these models included updating with forgetting, although for the data from Experiment 2, the model that used updating with full memory was the best-fitting model for almost as many participants. Recall that in Experiment 2, response repetitions/switches coincided with dimension repetitions/switches, so that there may have been ‘cross-talk’ between the two. The relatively good performance of the full-memory updating rule may be a consequence of such cross-talk; alternatively, it may be related to the explicit dimension (color vs. orientation) discrimination task in Experiment 2, which may have caused the memory of responses on previous trials to decay more slowly, compared to the simple-detection (target-present vs. -absent) task used in Experiments 1 and 3.

Finally, for the dimension-based updating, the best-fitting models differed among experiments. In Experiment 1, no version of dimension-based updating improved the fits sufficiently to motivate the extra parameter(s). For Experiments 2 and 3, by contrast, the various rate-based updating models most frequently provided the best account of the data. Given that the dimension only varied between mini-blocks in Experiment 1 (rather than varying randomly within each block, as in Experiments 2 and 3), it is little surprising that dimension-based updating played no significant role in modeling the data from Experiment 1. With regard to Experiment 2, it is less clear why the ‘rate with decay’ model consistently outperformed the other two rate-based updating models, while the ‘binary rate’ and ‘weighted rate’ models performed better in Experiment 3. There are two important differences between Experiments 2 and 3. First, the former used a dimension discrimination task, the later a detection task. Also, there were no target-absent trials in Experiment 2, whereas 50% of the trials were 'target-absent' in Experiment 3, so that dimension-based updating would affect only half of the trials. This could perhaps explain why it was worth the extra parameter to have a longer memory than a single trial back for dimension-based updating in Experiment 2, but not in Experiment 3 (where the ‘binary rate’ model, with a memory of only a single trial back but one less parameter, most frequently provided the best fit to the data). The better performance of the ‘Rate with decay’, compared to the ‘weighted rate’, version of rate updating in Experiment 2 is harder to explain with the present design; it may well have to do with the use of a discrimination (rather than a detection) task or the absence of no-target trials, but this requires further investigation.

Overall, the results of this form of model comparison closely matched those of comparing models based on the average AIC values: the factor levels that show up most frequently in the list of the best-fitting models are identical to those with the lowest average AICs, with just one exception: the ‘binary rate’ level would be preferred over the ‘weighted rate’ for dimension-based updating in Experiment 3. Importantly both the ‘binary rate’ and ‘weighted rate’ updating rules involve updating of the evidence accumulation rate, even though they differ in that the ‘weighted rate’ rule has a memory of more than one trial back.

**Table A** Model comparison across individual participants

| Models | Exp. 1 | Exp. 2 | Exp. 3 |
| --- | --- | --- | --- |
| RT distribution model: DDM | 0 | 0 | 0 |
| RT distribution model: LATER | 12 | 12 | 12 |
| Without non-decision time | 1 | 2 | 1 |
| With non-decision time | 11 | 10 | 11 |
| Response: No update | 0 | 0 | 0 |
| Response: S0 with full memory | 1 | 5 | 1 |
| Response: S0 with decay | 10 | 7 | 10 |
| Response: Binary rate | 0 | 0 | 0 |
| Response: Rate with decay | 1 | 0 | 1 |
| Response: Weighted rate | 0 | 0 | 0 |
| Dimension: No update | 7 | 0 | 0 |
| Dimension: S0 with decay | 0 | 0 | 3 |
| Dimension: Binary rate | 0 | 1 | 7 |
| Dimension: Rate with decay | 2 | 11 | 0 |
| Dimension: Weighted rate | 3 | 0 | 2 |
