# Supplementary material for: Inter-trial effects in visual pop-out search: Factorial comparison of Bayesian updating models
Source: PLoS Comput Biol. 2018 Jul 30;14(7):e1006328. doi: 10.1371/journal.pcbi.1006328 (PMC6091979; doi:10.1371/journal.pcbi.1006328)
Supplement: S5 Text — (DOCX) [file pcbi.1006328.s005.docx]

## S5 Text: The role of the forgetting rule in the inter-trial effects

Our starting point updating rule, ‘S0 with full memory’, predicts that inter-trial effects would decrease in magnitude over the course of an experiment, as the precision of the prior increases and new evidence consequently has less of an effect. The ‘S0 with decay’ rule, on the other hand, does not make this prediction, and our model comparison results clearly show that this rule explains the data better. In this section, we tested whether the size of inter-trial effects do, in fact, decrease over time, in order to confirm whether this difference in predictions is the reason why the ‘S0 with decay’ rule performed better. We did this by splitting the sequence of trials in half for each participant and experiment and calculating inter-trial effects for repetition versus switch of the response-defining feature separately for the early and late half of each experiment (see Figure A). In each experiment inter-trial effects were, in fact, slightly larger in the second half, although these differences were not statistically significant (Exp. 1: F(1,11)=2.28, p=0.16, BF=0.46 ; Exp. 2: F(1,11)=3.00, p=0.11, BF=0.50; Exp. 3: F(1,11)=0.45, p=0.52, BF=0.39). This contradicts the predictions of the ‘S0 with full memory’ rule and explains why the ‘S0 with decay’ rule performed better.


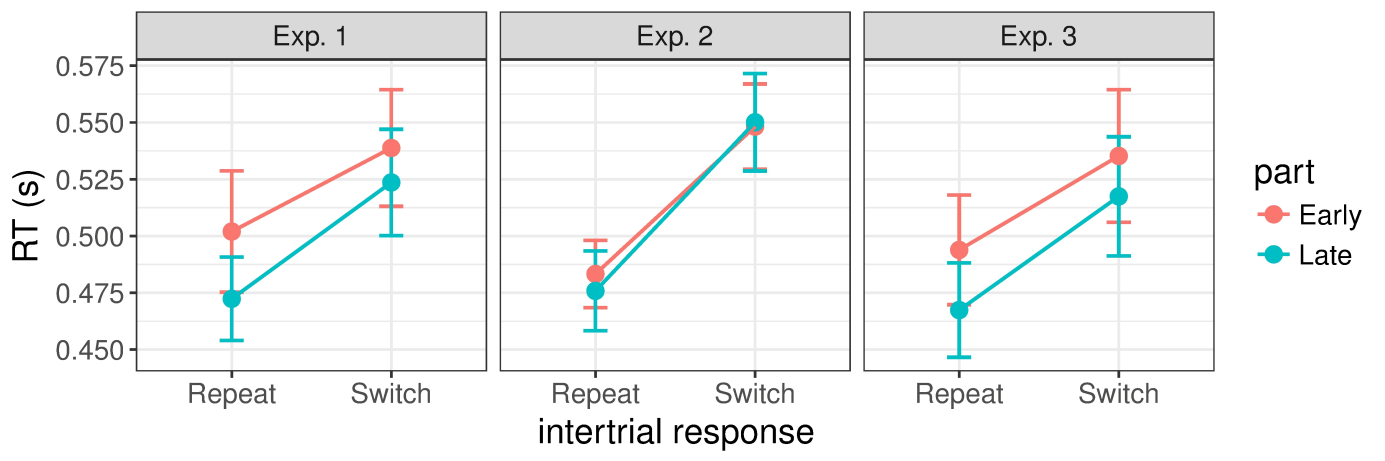


**Figure A** Effects of response feature repetition/switch on mean RTs in the early and late half of each experiment. Error bars show the standard error of the mean.
