# Supplementary material for: Inter-trial effects in visual pop-out search: Factorial comparison of Bayesian updating models
Source: PLoS Comput Biol. 2018 Jul 30;14(7):e1006328. doi: 10.1371/journal.pcbi.1006328 (PMC6091979; doi:10.1371/journal.pcbi.1006328)
Supplement: S6 Text — (DOCX) [file pcbi.1006328.s006.docx]

## S6 Text: Comparison of drift rate updating rules

As pointed out in the Discussion section, our two different drift rate updating rules with a memory of more than a single trial back – ‘rate with decay’ and ‘weighted rate’ – are in general quite similar, but make opposite predictions regarding two types of sequences: a sequence of repeats followed by a switch (i.e., {R, …, R, S}) and an equally long sequence of only switches ({S,…S,S}).

The ‘rate with decay’ rule predicts a higher rate after the {R, …, R, S} sequence compared to the {S, …, S, S} sequence, because, according to this rule, repeating a dimension always increases the rate on future trials regardless of dimension. The ‘weighted rate’ rule, by contrast, predicts that the rate should be particularly low after the {R, …, R, S} sequence, because each repetition means more weight is assigned to the repeated dimension and consequently less weight to the other dimension which will be the target dimension after the switch at the end.

Because the ‘rate with decay’ for dimension-based updating best explained the data in Experiment 2, while the ‘weighted’ rate rule best explained the data in Experiment 3, we tested whether RTs after the kind of sequences of dimension repetition and switch differ in the predicted way between these experiments. In particular, we compared RTs after sequences of two dimension repeats and one switch (RRS) with RTs after a sequence of three switches (SSS).


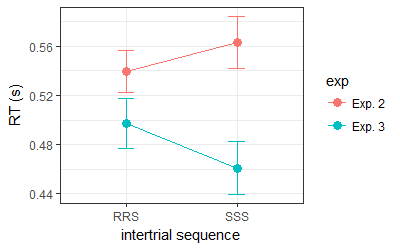


**Figure A** Mean RTs on switch trials at the end of different sequences of dimension repetitions (R) and switches (S) in Experiments 2 and 3. Error bars show the standard error of the mean.

Figure A shows the mean RTs at the end of these different sequences. In Experiment 2, RTs were significantly faster at the end of the RRS compared to the SSS sequence (t(11)=2.69, p<0.05), matching the prediction of the ‘rate with decay’ rule; in Experiment 3, by contrast, RTs were significantly faster at the end of the SSS compared to the RRS sequence (t(11)=2.3, p<0.05), following the prediction of the ‘(dimension-) weighted rate’ rule. Note that, in Experiment 2, the dimension was also the response-defining feature (RDF). Accordingly, the RTs for the different sequences of dimension repeats and switches could also have been influenced by starting-point updating based on the RDF. However, this is unlikely to explain the faster RTs at the end of RRS compared to SSS sequences, because the winning starting-point updating rule would make the opposite prediction. This implies that the effect of rate updating must have been greater than that of starting-point updating in Experiment 2, at least with regard to effects from more than one trial back (e.g., memory decay may have been slower for rate updates compared to starting-point updates). In addition, because the dimension was the RDF in Experiment 2, the rate updating based on dimension history in the winning model may partially reflect an effect of repetition/switch of the whole S–R link, rather than of just the dimension itself. This could explain the differential patterns seen in Experiment 2 vs. Experiment 3 (see main text for an elaboration of this argument).
