# Supplementary material for: Inter-trial effects in visual pop-out search: Factorial comparison of Bayesian updating models
Source: PLoS Comput Biol. 2018 Jul 30;14(7):e1006328. doi: 10.1371/journal.pcbi.1006328 (PMC6091979; doi:10.1371/journal.pcbi.1006328)
Supplement: S7 Text — (DOCX) [file pcbi.1006328.s007.docx]

**S7 Text: Model fits to RT distributions**

The LATER model in general fitted the RT distributions somewhat better than the DDM. In order to allow the reader to judge the quality of the fits for each model and the nature of the deviations, we here provide figures (Figures A-H) of individual subject RT distributions for each experiment, separately for color targets, orientation targets and target absent trials, and the LATER model and DDM fit to each distribution. We show these fits for models with no updating, because the effects of the updating rules only become relevant when taking trial history into account and do not improve the fit to the overall distributions, but with a non-decision time. The predictions of the DDM and the LATER model are in general quite similar, but the DDM tends to have a somewhat longer “tail”.


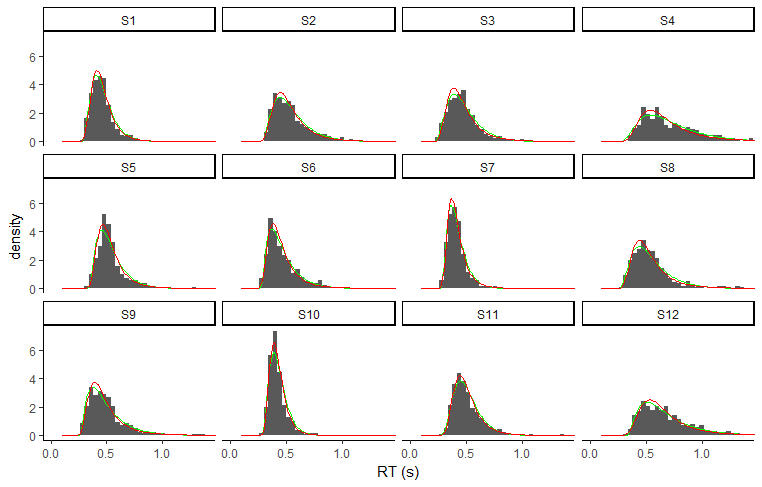


**Figure A** RT distributions for individual participants and model fits, without updating but with a non-decision time, for target absent trials in Experiment 1. The red line shows the fit of the LATER model while the green line shows the fit of the DDM.


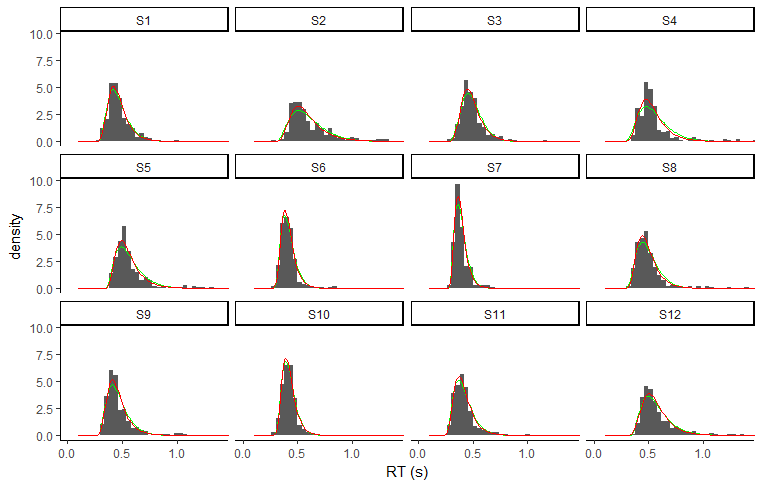


**Figure B** RT distributions for individual participants and model fits, without updating but with a non-decision time, for orientation target trials in Experiment 1. The red line shows the fit of the LATER model while the green line shows the fit of the DDM.


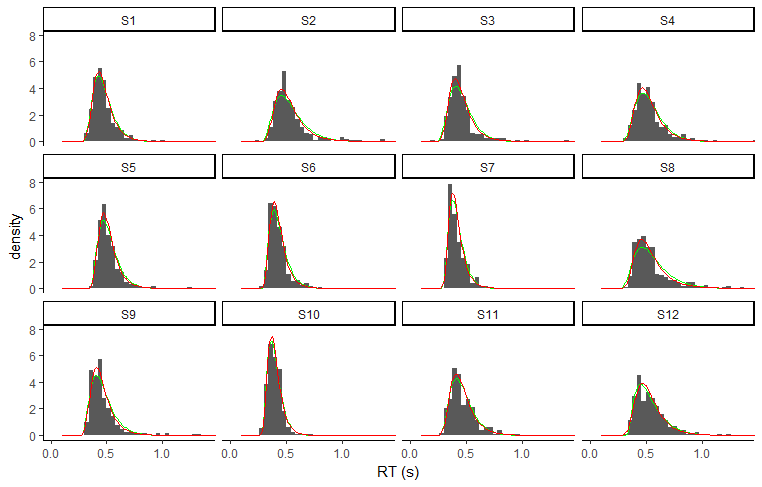


**Figure C** RT distributions for individual participants and model fits, without updating but with a non-decision time, for color target trials in Experiment 1. The red line shows the fit of the LATER model while the green line shows the fit of the DDM.


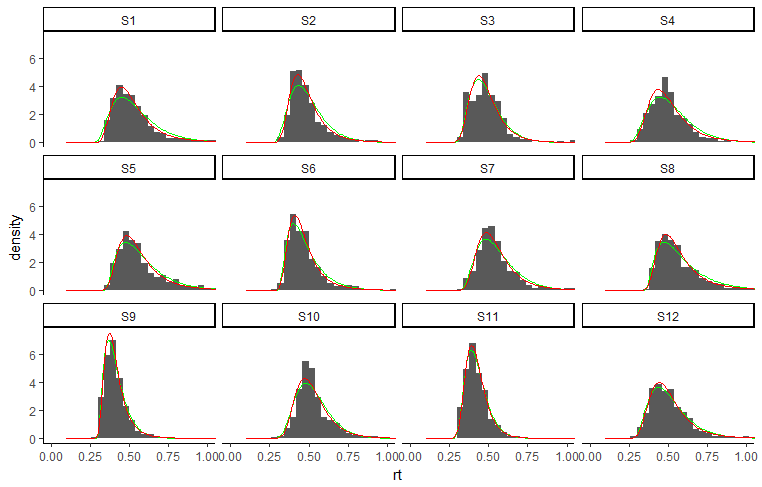


**Figure D** RT distributions for individual participants and model fits, without updating but with a non-decision time, for color target trials in Experiment 2. The red line shows the fit of the LATER model while the green line shows the fit of the DDM.


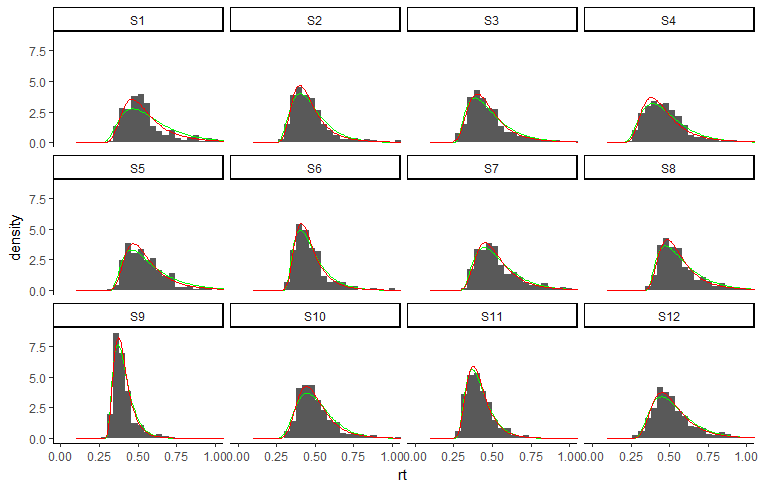


**Figure E** RT distributions for individual participants and model fits, without updating but with a non-decision time, for orientation target trials in Experiment 2. The red line shows the fit of the LATER model while the green line shows the fit of the DDM.


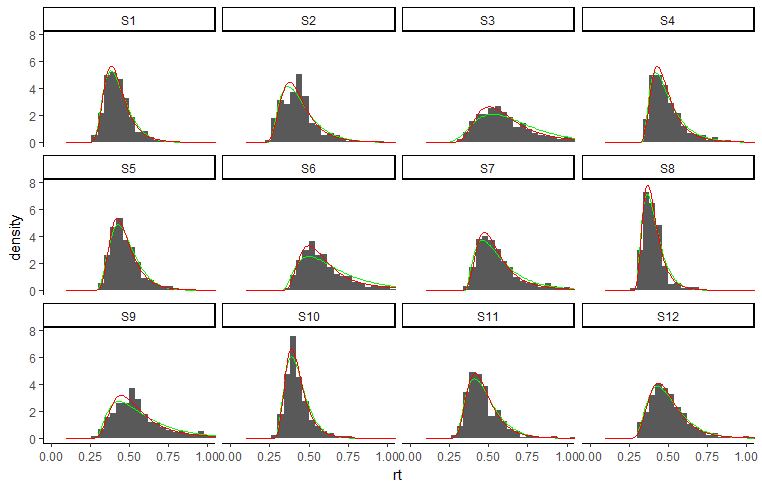


**Figure F** RT distributions for individual participants and model fits, without updating but with a non-decision time, for target absent trials in Experiment 3. The red line shows the fit of the LATER model while the green line shows the fit of the DDM.


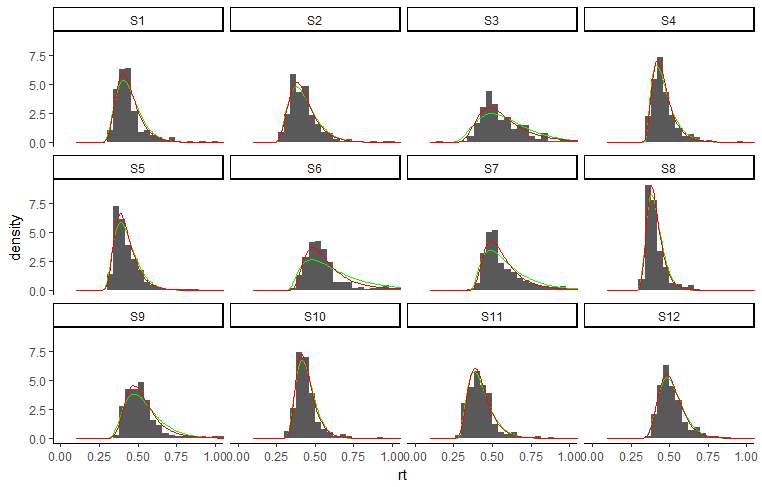


**Figure G** RT distributions for individual participants and model fits, without updating but with a non-decision time, for orientation target trials in Experiment 3. The red line shows the fit of the LATER model while the green line shows the fit of the DDM.


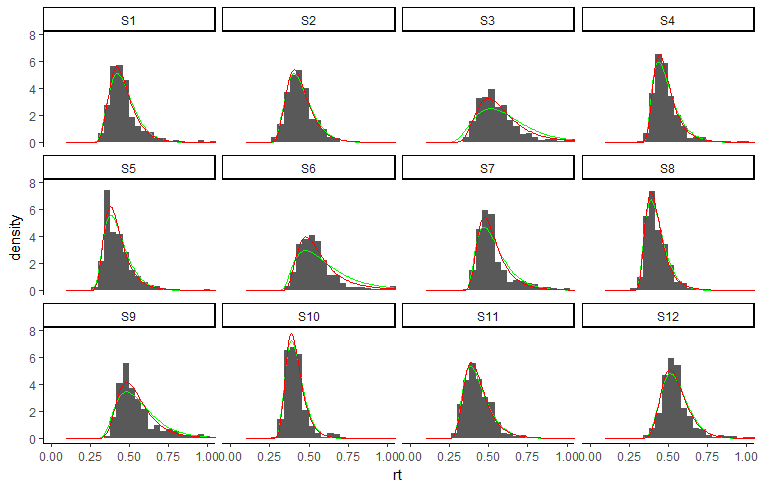


**Figure H** RT distributions for individual participants and model fits, without updating but with a non-decision time, for color target trials in Experiment 3. The red line shows the fit of the LATER model while the green line shows the fit of the DDM.
